# A Unique Case of Severe Anemia Secondary to Copper Deficiency in an Adult Patient

**DOI:** 10.7759/cureus.3636

**Published:** 2018-11-26

**Authors:** Saad Atiq, James M Mobley, Osman O Atiq, Mohammad O Atiq, Nikhil Meena

**Affiliations:** 1 Miscellaneous, University of Arkansas for Medical Sciences, Little Rock, USA; 2 Internal Medicine, University of Arkansas for Medical Sciences, Little Rock, USA; 3 Miscellaneous, St. Matthew's University, Little Rock, USA; 4 Internal Medicine, Memorial Sloan Kettering Cancer Center, New York, USA

**Keywords:** anemia, micronutrients, copper, zinc, hypocupremia

## Abstract

Anemia is a frequently encountered problem in the healthcare system. Common causes of anemia include blood loss, followed by impaired red blood cell production and red blood cell destruction. This case demonstrates the need for cognizance of the less frequent causes of anemia.

A 27-year-old male with a history of traumatic brain injury and quadriplegia with chronic respiratory failure on home ventilator support presented to the emergency department with dyspnea and no bowel movements for three days. The patient received nutrition via percutaneous endoscopic gastostromy (PEG) tube. He was hypotensive with a mean arterial pressure (MAP) of 54 mm/Hg. There was no evidence of acute or ongoing blood loss. Initial lab data revealed hyperkalemia (K+ 6.1), severe anemia (Hb 1.5 g/dL), leukopenia (2.53 K/uL), neutropenia (ANC 700), and normal platelets. Peripheral smear revealed leukopenia with absolute neutropenia, marked anemia with anisopoikilocytosis with rare dacrocytes but no evidence of schistocytes. He responded to transfusion with improvement in hemoglobin from 1.5 to 9.1 within 24 hours. There was no evidence of hemolysis or vitamin deficiency. Ferritin and triglyceride levels were ordered to rule out hemophagocytic lymphohistiocytosis (HLH). Ferritin was elevated at 6506 ng/mL and triglycerides were 123 mg/dL. Soluble IL-2 receptor level was sent and found to be significantly elevated; however, this was felt to be more likely secondary to infection and inflammation, as the patient had no other clinical features of HLH, apart from cytopenias. Zinc supplementation was part of his wound care regimen. Copper levels were <10 ug/dL (normal: 70-140). Zinc supplements were stopped, and the patient was started on copper supplementation. At his three month follow-up clinic appointment, his anemia and leukopenia had resolved.

Micronutrient deficiency is a potential cause of anemia, especially in a risk population and must be considered, as it is often easily correctible.

## Introduction

Anemia is a frequently encountered problem in the healthcare system. In the United States, anemia prevalence was found to be nearly six percent over a 10 year period, with various factors such as age, gender, and race playing a role [1]. Common causes of anemia include blood loss, impaired red blood cell production, and red blood cell destruction [2]. While these are the most common causes, other potential etiologies include diet, hormonal imbalance, pregnancy, mineral deficiency, and illness. This case demonstrates the need for cognizance of the less frequent causes of anemia. 

## Case presentation

A 27-year-old male with a history of traumatic brain injury and quadriplegia, with chronic respiratory failure on home ventilator support, presented to the emergency department with increased work of breathing and no bowel movements for three days. The patient was bed-bound, nonverbal, and received nutrition via percutaneos endoscopic gastostromy (PEG) tube. The patient was found to have long-standing anemia with an average hemoglobin (Hb) of 9 g/dL and leukopenia for 2 years. He was hypotensive with a mean arterial pressure (MAP) of 54 mm/Hg. The rest of his physical exam was unremarkable, and there was no evidence of acute or ongoing blood loss. Chest X-ray revealed a right pleural effusion. A central venous line was placed, and the patient was started on vancomycin and cefepime for presumed sepsis. Initial lab data revealed hyperkalemia (K+ 6.1), severe anemia (Hb 1.5 g/dL), leukopenia (2.53 K/uL), neutropenia (ANC 700), and normal platelets. He was also found to be have acute kidney injury with creatinine (Cr) of 1.5 (mg/dL), and anion-gap metabolic acidosis with a lactate of 7.0 (mmol/L). The patient required norepinephrine support for septic shock. Peripheral smear revealed leukopenia with absolute neutropenia, marked anemia with anisopoikilocytosis, with rare dacrocytes but no evidence of schistocytes. He responded appropriately to blood transfusion with improvement in hemoglobin from 1.5 to 9.1 within 24 hours. He did not require further transfusion during hospitalization.

Investigation of the profound anemia

Evaluation for hemolysis failed to reveal an etiology. His vitamin levels (cobalamine and folate) were within the normal range. He had no personal or family history of hemoglobinopathy, and hemoglobin electrophoreses was normal. Ferritin and triglyceride levels were ordered to rule out hemophagocytic lymphohistiocytosis (HLH). Ferritin was elevated at 6506 ng/mL and triglycerides were 123 mg/dL. Soluble IL-2 receptor level was sent and found to be significantly elevated; however, this was felt to be more likely secondary to infection and inflammation, as the patient had no other clinical features of HLH, with the exception of cytopenias. Further questioning revealed significant consumption of zinc supplements as part of his wound care regimen. This necessitated an evaluation of micronutrients including copper. The copper levels were found to be <10 ug/dL (normal: 70-140). The patient’s hypotension improved with management of sepsis. His renal function normalized. Zinc supplements were stopped, and the patient was started on copper supplementation. At his three month follow-up clinic appointment, his anemia and leukopenia had resolved. 

## Discussion

This case presents a unique perspective given the severity of the patient’s anemia. While there are several more common causes of anemia, particularly in a certain at-risk population, micronutrient deficiencies must be considered in the differential diagnosis. Copper deficiency has a rare, but well-documented association with anemia and neutropenia, particularly in children receiving supplemental nutrition. Hypocupremia was found in marasmic infants fed with calorie-rich milk diets [3]. Its incidence in adults has been less well characterized. Neutropenia has been found to be both an early manifestation and a sensitive indicator of treatment of copper deficiency. Other signs of hypocupremia include hair and skin changes, neurologic and cardiovascular disorders, and skeletal abnormalities [3-4]. Although anemia and neutropenia are seen, bone marrow sampling of these patients revealed normal levels of progenitor cells, indicating a role for copper in the maturation of precursor cells of the bone marrow [5]. Similarly, hypocupremia has been shown to affect rates of iron absorption, mobilization, and utilization. The resultant hypochromic, microcytic anemia is refractory to iron supplementation alone, supporting concurrent copper supplementation [6].

Risk factors for copper deficiency include history of gastrointestinal surgery, zinc supplementation, malabsorption, and parenteral nutrition [7]. A rarer, yet still documented cause is copper deficiency secondary to a ketogenic diet [8]. Occult forms of zinc supplementation leading to hypocupremia have also been noted, such as the use of zinc-containing dental adhesive [9]. Disorders in which there is a deficiency of ceruloplasmin such as Wilson’s disease may also result in copper deficiency, as greater than 90% of copper stored in the body is bound to ceruloplasmin [4]. Bone marrow changes in copper deficiency include cytoplasmic vacuoles in erythroid and myeloid precursors, with sea-blue histiocytes, and anisopoikilocytosis [10]. Other marrow findings include increased early granulocytes, megaloblastic red blood cells, and increased sideroblasts [11]. These characteristic findings are useful in differentiating between copper deficiency and myelodysplastic syndrome (MDS). In our case, only the megaloblastic red blood cells were seen along with anisopoikilocytosis, demonstrating variability in the presentation of copper deficiency. 

**Figure 1:**
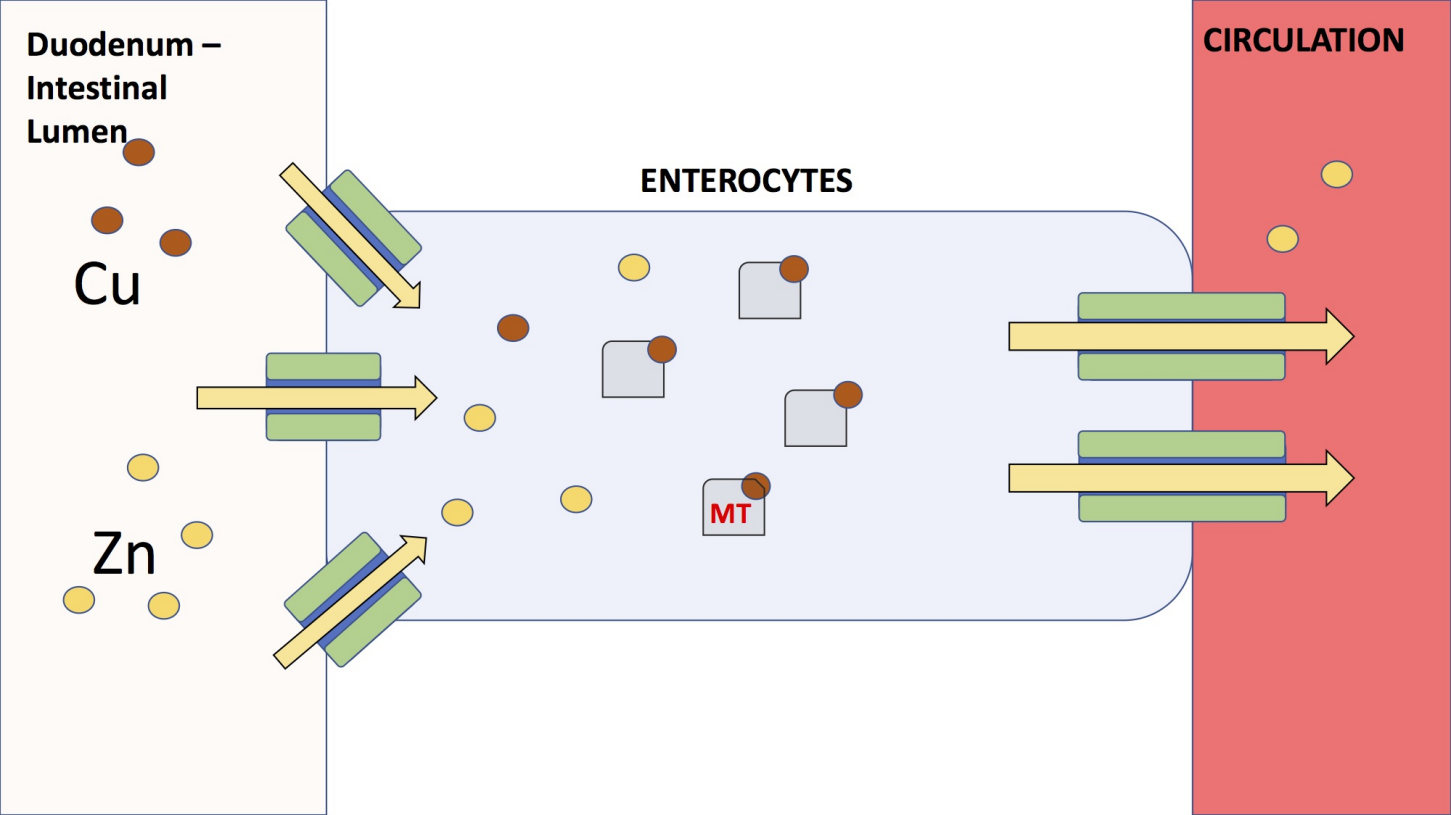
Copper-zinc metabolism. Copper and zinc are primarily absorbed at the duodenal surface. Zinc and copper are transported into the cell, where zinc is typically bound by metallothionein (MT), and upregulates its synthesis. In the presence of copper and zinc, MT preferentially binds copper. Thus, in the setting of zinc supplementation, there is excess MT production. This causes more copper to be bound and excreted in the stool, instead of getting absorbed into hepatic circulation.

The aforementioned patient had two key risk factors for hypocupremia: parenteral nutrition and zinc supplementation. Copper absorption normally occurs in the duodenum (Figure 1). In patients receiving long-term parenteral nutrition without copper supplementation, the risk for copper deficiency is greatly increased. Likewise, zinc supplementation may cause copper deficiency through a different mechanism of action. Zinc is usually added in quadriplegic patients to aid in both prevention and healing of decubitus ulcers. However, zinc can cause increased production of metallothionein (MT) by intestinal enterocytes, leading to increased copper excretion and decreased responsiveness to erythropoietin stimulating agents [12-14].

Both these actions can lead to severe anemia. Once the copper deficiency is identified as the source of anemia, it is easily corrected with dietary copper supplementation thereby stopping zinc supplements.

## Conclusions

This is the case of a 27-year-old male with a history of traumatic brain injury and quadriplegia, with chronic respiratory failure on home ventilator support, suffering from zinc toxicity induced hypocupremia with resultant anemia. Micronutrient deficiency is a potential cause of anemia, especially in risk population and must be considered, as it is often easily correctible.
